# A survey of health status of healthcare providers in a square cabin hospital during the new corona omicron outbreak: A cross-sectional study

**DOI:** 10.3389/fpsyg.2022.1028631

**Published:** 2022-11-11

**Authors:** Haoyu Pei, Xiaoqin Gan, Fei Guo, Qiuping Wu, Ding Liu, Zhouzhou Li, Ping Lan, Lili Zhang, Hong Yan

**Affiliations:** ^1^Department of Anesthesiology, Army Medical Center of PLA, Chongqing, China; ^2^Department of Medical Research, Army Medical Center of PLA, Chongqing, China; ^3^Department of Cardiology, Xinqiao Hospital, Army Medical University, Chongqing, China; ^4^Department of Infection, Army Medical Center of PLA, Chongqing, China; ^5^Department of Neurology, Army Medical Center of PLA, Chongqing, China

**Keywords:** SARS-CoV-2, medical staff, healthcare providers, mental health, somatic symptoms

## Abstract

**Background:**

The coronavirus omicron variant outbroke in early 2022 in Shanghai. Although previous studies indicated that long working hours in a square cabin hospital might increase the risk of mental health among frontline healthcare providers, few studies have investigated whether the mental health risk could be reduced among well-trained professionals following the new guidelines.

**Objective:**

This study aimed to investigate the health situation of frontline healthcare providers in Shanghai square cabin during the omicron variant circulation.

**Methods:**

An online survey was used to evaluate those healthcare providers working in the square cabin hospitals from March 1, 2022, to May 31, 2022. The first online survey was conducted and emailed to the health providers on April 1. The second survey was conducted and sent to the nonrespondents on May 31. Overall, 142 frontline healthcare providers completed the online survey. Their mental health was assessed by the Insomnia Severity Index Scale, the Generalized Anxiety Disorder Scale, the Patient Health Questionnaire-9, and the Psychological Resilience Scale. We estimated multiple clinical systems and identified factors associated with those symptoms among participants. Multivariable logistic regression models were used to assess the risk factors of these symptoms.

**Results:**

Overall, 66.20%, 45.07%, and 27.46% of frontline healthcare providers in Shanghai City reported symptoms of insomnia, depression, and anxiety, respectively. In addition, the most common symptoms included dry eyes (57.75%), lumbar muscle strain (47.18%), dry mouth (35.92%), itching (31.69%), headache (29.58%), and sore throat (28.87%) among the frontline healthcare providers. There was no statistical difference in symptoms by gender, age, personnel category, or job position (*p* > 0.05).

**Conclusion:**

In the case of an unexpected pandemic, the mental health of healthcare providers is not optimistic. This situation still exists more than 2 years after the global outbreak of the COVID-19 pandemic. Therefore, the physical and mental health of long-term healthcare providers working in a square cabin hospital still needs monitoring.

## Introduction

The COVID-19 pandemic outbreaks in 2019. China has taken strict and effective public health measures to contain the pandemic, such as encouraging people to wear protective masks, self-isolation, canceling mass gatherings, etc. Pandemic prevention and control have become the norm. However, sporadic cases in some areas face more stringent measures to prevent and control the pandemic. The establishment of the square cabin hospital can facilitate the rapid expansion of admission and interception of the source of infection. Previous studies established the significance of square cabin hospitals in China. However, healthcare providers faced many challenges in working in a square cabin hospital, such as limited environmental conditions, huge patient volume (about 500 patients per shift), dense infected population, rapid turnover, and complex demographic characteristics of patients. It poses many challenges for frontline medical staff as well.

Previous studies indicated that long-term work in a square cabin hospital might increase the risk of mental health issues among the frontline healthcare providers, such as anxiety, depression, and insomnia. This phenomenon was observed as early as the severe acute respiratory syndrome (SARS) outbreak in 2003 and the Middle East respiratory syndrome (MERS) outbreak in 2015. However, healthcare workers neglect mental health issues in response to an outbreak. Due to the first time COVID-19 outbreak, scientists, healthcare providers, government workers, and ordinary citizens lacked awareness of this new virus. A population of 69% experienced above-moderate psychological distress (Rahman et al., 2021). Panic is one of the reasons for the massive pressure on frontline healthcare providers. Other risk factors for mental health issues include but are not limited to high-intensity anti-epidemic work and complex environments. In China, the incidences of anxiety, depression, and insomnia among the frontline healthcare providers who were long-term working in square cabin hospitals ranged from 25% to 58%, 29% to 54%, and 34% to 36%, respectively. These mental health problems are much more serious than non-frontline healthcare workers (Zhang et al., 2022). Studies have shown progressively higher anxiety levels in frontline healthcare workers over time, with heavy nursing tasks, risk of infection, and isolation as common risk factors (Luo et al., 2020). On 2020 March 24, the first Chinese Clinical Guidance for COVID-19 Pneumonia Diagnosis and Treatment was published. Up to date, the characteristics and treatments of coronavirus Omicron have been carefully described in the latest guidelines. The pandemic in China is developing in a positive direction. The pandemic is under control, and the psychological state of frontline medical staff was well-responded in the early stages of the pandemic. At the same time, there was the occasional localized worsening of the pandemic, changes in work intensity, medical resource mobilization, and changing Working mode. Furthermore, frontline medical staff will be isolated from their families in Fangzhan hospitals with strict isolation policies, reducing their social support. All these factors may lead to loneliness and helplessness, triggering mental health problems, which in turn may reduce the motivation of healthcare workers and negatively affect the quality of care (Tawfik et al., 2019). Therefore, the mental health of frontline medical staff performing medical rescues in off-site locations deserves serious attention (Wu et al., 2022).

### General framework about Mental Health, Environment, Event, and Technology

Because mental health issues among healthcare providers had not been taken seriously during the outbreaks, high levels of stress, depression, and anxiety were still observed among some frontline healthcare providers. This framework was investigated and named MEET (Mental Health, Environment, Event, and Technology) by Ye in 2021 (Brainin et al., 2018). However, in this framework, environmental factors were not thoroughly investigated, and they only considered COVID-19. Therefore, it is essential to identify and monitor those healthcare providers with a high risk of developing mental health issues and carry out early psychological and psychiatric interventions. This survey aims to understand the risk factors of mental health issues, including somatic symptoms and psychological and sleep conditions among the frontline healthcare providers working long-term in square cabin hospitals, providing a basis for the prevention of psychological problems in these healthcare workers and the development of interventions.

## Materials and methods

### Study population

The study was conducted from April 1, 2022, to May 31, 2022. We used an online survey *via* the WeChat-based “Questionnaire Star” program to collect data (Li et al., 2019). According to the relevant literature, the sample size is known to have an incidence rate of 69%. The tolerance error *δ* is taken as 0.1 with *α* of 0.05 (95% CI), *Z*_α/2_ = 1.96 substituted into the formula, and an attrition rate of 0.1 is expected to require at least 93 cases of the sample. In this study, 142 subjects participated in the survey, and all the questionnaires were completed following the requirements. A total of 142 valid questionnaires were collected, which was greater than the expected sample size and could meet the needs of the study.

### Ethical considerations

The STROBE (Strengthening the Reporting of Observational Studies in Epidemiology) guidelines for observational studies were followed. The study was carried out following the guidelines of the Declaration of Helsinki and Good Clinical Practice. The subjects in this study were surveyed only after giving full informed consent and were informed that a refusal of the survey would not have any effect and that no specific names or units were involved in the questionnaire, which would not reveal any privacy of the study subjects. The study protocol was approved by the Army Medical Center of PLA in China [permission no. 2022(154)].

### Development of questionnaire and data collection

The survey questionnaire commenced with a detailed written description of each attribute and its levels, followed by a set of questions. Questions regarding socio-demographic characteristics (e.g., age, gender, and marital status) were asked. The mental health scenarios were generated using a homemade somatic symptom scale, Insomnia Severity Index (ISI), Generalized Anxiety Disorder Scale (GAD-7), Patient Health Questionnaire (PHQ-9), and Psychological Resilience Scale (Connor-Davidson Resilience Scale, CD-RISC).

A pilot test involving 20 healthcare providers from the Army Medical Center of PLA was conducted to modify the questionnaires (the data from these 20 patients were not included in the data analysis of the primary investigation). Epidemiologists from the Army Medical Center of PLA were consulted to evaluate the quality of the questionnaire.

The survey was conducted anonymously. Inclusion criteria for this study included: (a) age over 18 years; (b) At the time of the survey, healthcare providers from all regions of China were working in the square for at least 2 weeks; (c) Ability to use Chinese language for internet access, reading, and writing. Exclusion criteria: (a) history of psychiatric disorders; (b) sleep quality affected by adverse personal events; (c) taking sleep-regulating medications (sedatives and hypnotics); (d) incomplete data from the online questionnaire. All participants read the questionnaire instructions and informed consent. Before the questionnaire, they were informed of the study protocols, survey content, and purpose. The whole questionnaire took about 5 min to complete. The questionnaire star was set up so that each question needed to be answered before submission, and each subject could only submit once to prevent duplicate submissions. We excluded participants in the data analysis if they reported extreme answers, defined as having the same response to all survey questions.

### Measurements of mental health issues

In the survey questionnaire, we measured somatic symptoms, insomnia, anxiety, depressive symptoms, and psychological resilience using a homemade somatic symptom scale, Insomnia Severity Index (ISI), Generalized Anxiety Disorder Scale (GAD-7), Patient Health Questionnaire (PHQ-9), and Psychological Resilience Scale (Connor-Davidson Resilience Scale, CD-RISC). Specifically, the homemade somatic symptom survey scale includes 46 questions. The results were summarized into five questions on the respiratory tract, digestive tract, skin mucosa, eye discomfort, and others, each containing yes or no two answers. ISI was used to assess insomnia, which includes seven entries using a five-point scale from 0 to 4. Participants rate the items based on the first 2 weeks. The total scores are 0–7 for no insomnia, 8–14 for mild insomnia, 15–21 for moderate insomnia, and 22–28 for severe insomnia (Morin et al., 2011). In this study, the Cronbach coefficient was 0.90GAD-7 (Spitzer et al., 2006), which indicated an effective tool for screening generalized anxiety disorder (Spitzer et al., 2006). The screening for generalized anxiety disorder includes seven entries using a four-point scale from 0 to 3. The total score range is 0–21, and a higher total score indicates very severe anxiety. Specifically, a score of 5–9 indicates mild anxiety, and ≥10 indicates moderate to severe anxiety symptoms (Plummer et al., 2016). The Cronbach’s coefficient in this study was 0.93.PHQ-9 (Kroenke et al., 2001) is used to assess depressive symptoms (Kroenke et al., 2001). A four-point scale of 0 to 3 is used in PHQ-9. The total score ranges from 0 to 27. The higher the total score means, the more severe the depression. Specifically, a total score of 0–4 indicates no depressive tendency, 5–9 indicates mild depressive tendency, 10–14 indicates moderate depressive tendency, 15–19 indicates moderate to severe depression, and 20 or more indicates severe depression (Kroenke et al., 2001). The reliability and validity of the simplified Chinese version of the PHQ-9 have been demonstrated (Wang et al., 2014). In this study, the scale showed good internal consistency (Cronbach’s coefficient of 0.91 for frontline healthcare providers and 0.89 for the general population). The Psychological Resilience Scale (Connor-Davidson Resilience Scale, CD-RISC; Cheng et al., 2020) measures psychological resilience, including 25 items using a five-point scale from 0 to 4. The total score ranges from 0 to 100; the higher the score, the better psychological resilience. In this study, Cronbach’s coefficient of this scale was 0.95. The same scale was applied to regular medical staff to obtain the relevant normative parameters (Hou et al., 2020; Liang et al., 2020; Zhang et al., 2020).

### Covariates

Demographic information included gender, age, marriage, and job title. Other covariates included marriage status, weekly working hours, night shifts per week, and the number of patient care cases per day.

### Statistical analyses

Count data were expressed as frequencies (percentages). They were compared using the Chi-square test for categorical variables and the *t*-test for continuous variables between groups. When the measurement data did not conform to the normal distribution, the median (interquartile spacing) was used to describe the data, and the nonparametric test was used to compare groups. Adjusted odd ratios (ORs) and 95% confidence intervals (CIs) of outcomes at a given time were compared across the different groups using multivariate logistic regression models. The multivariate models were adjusted for covariates. All analyses used SPSS 25.0 with *p* < 0.05 as statistical significance.

## Results

A total of 142 frontline healthcare providers completed the survey. Table 1 presents the socio-demographic characteristics of frontline healthcare providers. The participants in the study tend to be female, married, young, and middle-aged in more enormous proportions than the general population.

**Table 1:** General information.

Entry	Description
Gender	
Male	33 (23.24)
Female	109 (76.76)
Age (years)	
25–29	31 (21.83)
30–35	52 (36.62)
36–40	32 (22.54)
41–45	21 (14.79)
>45	6 (4.23)
Marriage	
Unmarried	39 (27.46)
Married	96 (67.61)
Divorce	7 (4.93)
Job title	
Director/Chief Nurse	20 (14.08)
Physicians	27 (19.01)
Nurse	95 (66.90)
Weekly working hours in the cabin	
12-20 h	15 (10.95)
21-28 h	96 (70.07)
>28 h	26 (18.98)
Number of night shifts per week (0:00–4:00; 4:00–8:00; 20:00–24:000)	
1–2	22 (15.49)
3–4	111 (78.17)
5–6	9 (6.34)
Number of patient care cases per day	
<300	46 (32.39)
300–399	32 (22.54)
400–499	34 (23.94)
500–700	30 (21.13)

The symptoms of each system are analyzed and described in Tables 2–6, where the response rate is used to compare the relative selection ratio of each symptom. The prevalence rate is used for the prevalence of the selection of a particular item. Specifically, sore throat (28.87%), stuffy and runny nose (21.83%), and cough (17.61%) were the top three prevalent symptoms in the respiratory system. Dry mouth (35.92%), constipation (29.58%), diarrhea (25.35%), and anorexia (25.35%) were the top three prevalent symptoms in the gastrointestinal system. In the skin system, the top three prevalent symptoms are pruritus (31.69%), peeling (28.87%), and allergic dermatitis (21.83%). In the visual system, the top three prevalent symptoms were dry eye (57.75%), dark eye circles (33.80%), and eye swelling (14.79%). Moreover, headache (29.58%), lumbar muscle strain (47.18%), and falling (24.65%) were the top three prevalent symptoms of other discomforts.

**Table 2:** Respiratory symptoms.

Item	Response		Prevalence rate (*n* = 142)
	*n*	Response rate	
Breathing difficulties	11	5.58%	7.75%
Chest tightness	21	10.66%	14.79%
Cough	25	12.69%	17.61%
coughing up sputum	10	5.08%	7.04%
Sore Throat	41	20.81%	28.87%
Stuffy and runny nose	31	15.74%	21.83%
A diminished sense of smell	2	1.02%	1.41%
Fever	3	1.52%	2.11%
Other	53	26.90%	37.32%
Aggregate	197	100%	138.73%

Goodness-of-fit test: *χ*^2^ = 111.421; *p* < 0.001.

**Table 3:** Gastrointestinal symptoms.

Digestive tract symptoms	Response		Prevalence rate (*n* = 142)
	*n*	Response rate	
Acid Reflux	16	6.30%	11.27%
belching	16	6.30%	11.27%
anorexia	36	14.17%	25.35%
bloating	32	12.60%	22.54%
Abdominal pain	4	1.57%	2.82%
Diarrhea	36	14.17%	25.35%
Constipation	42	16.54%	29.58%
Dry mouth	51	20.08%	35.92%
bitterness in the mouth	14	5.51%	9.86%
Other	7	2.76%	4.93%
Aggregate	254	100%	178.87%

Goodness-of-fit test: *χ*^2^ = 90.646; *p* < 0.001.

**Table 4:** Skin mucosal symptoms.

Skin and mucous membrane symptoms	Response		Prevalence rate (*n* = 142)
	*n*	Response rate	
Pruritus	45	19.48%	31.69%
Pimples	20	8.66%	14.08%
Herpes	5	2.16%	3.52%
Peeling	41	17.75%	28.87%
Skin and mucous membrane bleeding	4	1.73%	2.82%
Feeling abnormal	8	3.46%	5.63%
Allergic Dermatitis	31	13.42%	21.83%
Skin breakdown and infection at the site of the mask	26	11.26%	18.31%
Mouth ulcers	18	7.79%	12.68%
Other	33	14.29%	23.24%
Aggregate	231	100%	162.68%

Goodness-of-fit test: *χ*^2^ = 83.329; *p* < 0.001.

**Table 5:** Ocular symptoms.

Item	Response		Prevalence rate (*n* = 142)
	*n*	Response rate	
Dry eye	82	36.61%	57.75%
Weeping	25	11.16%	17.61%
Photophobia	5	2.23%	3.52%
Eye swelling	21	9.38%	14.79%
Dark Eye Circles	48	21.43%	33.80%
Bleeding from the eyes	1	0.45%	0.70%
Elevated intraocular pressure	4	1.79%	2.82%
Darkness in the eyes	1	0.45%	0.70%
Stye	8	3.57%	5.63%
Conjunctivitis	7	3.13%	4.93%
Other	22	9.82%	15.49%
Aggregate	224	100%	157.75%

Goodness-of-fit test: *χ*^2^ = 303.116; *p* < 0.001.

**Table 6:** Overall symptoms.

Other discomfort	Response		Prevalence rate (*n* = 142)
	*n*	Response rate	
Headache	42	25.93%	29.58%
Giddiness	9	5.56%	6.34%
Falling	35	21.60%	24.65%
Lumbar muscle strain	67	41.36%	47.18%
Other	9	5.56%	6.34%
Aggregate	162	100%	114.08%

Goodness-of-fit test: *χ*^2^ = 73.802; *p* < 0.001.

The PHQ-9 and GAD-7, PHQ-9, and CD-RISC were used to assess insomnia, anxiety, depression, and psychological resilience. The results are shown in Tables 7, 8. Specifically, the prevalence of insomnia, anxiety, and depression was 66.2%, 27.46%, and 45.07%, respectively. The mean of each measurement score of all respondents was 10.95 ± 6.52 (ISI), 3.03 ± 3.11 (GAD-7), 4.91 ± 4.24 (PHQ-9), and 67.58 ± 14.28 (CD-RISC).

**Table 7:** Distribution of insomnia, anxiety, and depression among frontline medical personnel.

Projects	Description (*n* = 142)
Insomnia severity index	
No insomnia	48 (33.80)
Mild insomnia	53 (37.32)
Moderate insomnia	30 (21.13)
Severe insomnia	11 (7.75)
GAD-7 anxiety and depression screening	
No anxiety	103 (72.54)
Mild anxiety	32 (22.54)
Moderate anxiety	6 (4.23)
Moderate to severe anxiety	1 (0.70)
Depression screening scale	
No depression	78 (54.93)
Mild depression	45 (31.69)
Moderate depression	16 (11.27)
Moderate to severe depression	1 (0.70)
Major depression	2 (1.41)
Psychological resilience	67.58 ± 14.33

**Table 8:** Comparison of frontline medical staff and control group.

	ISI		GAD-7		PHQ-9		CD-RISC	
	Number of cases	Score	Number of cases	Score	Number of cases	Score	Number of cases	Score
Frontline medical staff	142	10.95 ± 6.52	142	3.03 ± 3.11	142	4.91 ± 4.24	142	67.58 ± 14.28
Medical workers (standing models)	927	6.70 ± 5.76	761	4.75 ± 4.54	761	6.33 ± 5.59	1,472	68.40 ± 14.86
Difference estimate (95% CI)	4.250 (3.115, 5.385)		−1.720 (−2.325, −1.115)		−1.420 (−2.223, −0.617)		−0.820 (−3.288, 1.648)	
*z*-value	7.341		−5.575		−3.468		−0.651	
Value of *p*	**<0.001**		**<0.001**		**0.001**		0.515	

The bold value represents statistically significant.

In addition, ISI, GAD-7, PHQ-9, and CD-RISC control normative data from a survey of medical personnel during the outbreak (Hou et al., 2020; Liang et al., 2020). The results showed that ISI (*Z* = 7.341, *p* < 0.001), GAD-7 (*Z* = −5.575, *p* < 0.001), PHQ-9 (*Z* = −3.468, *p* < 0.001), and CD-RISC (Z = −0.651, *p* = 0.515), as shown in Table 8.

Influence factors were analyzed for ISI, GAD-7, and PHQ-9, where the *p* < 0.05 results were as follows. Specifically, the results of multivariate logistic regression models for the associations between the risk factors and each psychological outcome are presented in Tables 9A–9F. Overall, patients with older age (above 40 years old) and a higher score of psychological resilience had the lowest statistically significant odds of insomnia (OR = 0.27, 95% CI = 0.082–0.889 OR = 0.97, 95% CI = 0.95–0.99). In addition, participants with a higher score of psychological resilience also had statistically significant lower odds of anxiety (GAD-7 scores) and depression (PHQ-9 scores; OR = 0.95, 95% CI = 0.93–098; OR = 0.96, 95% CI = 0.93–098, respectively). However, participants’ socio-demographic factors had no significant change in the odds of anxiety and depression.

**Table 9A:** Analysis of the influencing factors of ISI.

	Total (*n* = 142)	No insomnia (*n* = 48)	Have insomnia (*n* = 94)	Statistical quantities	*p*
Age (years)				8.435	**0.038**
25–29	31 (21.83)	6 (19.35)	25 (80.65)		
30–35	52 (36.62)	20 (38.46)	32 (61.54)		
36–40	32 (22.54)	8 (25.00)	24 (75.00)		
>40	27 (19.01)	14 (51.85)	13 (48.15)		
Psychological resilience score	67.58 ± 14.33	71.81 ± 15.65	65.41 ± 13.18	2.565	**0.011**

The bold value represents statistically significant.

**Table 9B:** Analysis of the influencing factors of ISI.

Variable	Estimate	SE	*z*	Wald	*p*	OR (95% CI)
(Intercept)	3.307	1.035	3.194	10.204	**0.001**	
Age (years)						
25–29	ref					
30–35	−0.807	0.547	−1.476	2.177	0.14	0.446 (0.153, 1.303)
36–40	−0.195	0.622	−0.313	0.098	0.754	0.823 (0.243, 2.785)
>40	−1.309	0.608	−2.153	4.637	**0.031**	0.270 (0.082, 0.889)
Psychological resilience score	−0.029	0.014	−2.075	4.307	**0.038**	0.971 (0.945, 0.998)

The bold value represents statistically significant.

**Table 9C:** Analysis of influencing factors of GAD-7.

	Total (*n* = 142)	No anxiety (*n* = 103)	Have anxiety (*n* = 39)	Statistical quantities	*p*
The degree of stress caused by unfamiliarity with the workflow of the other cabin hospital	40.07 ± 26.04	37.14 ± 25.76	47.82 ± 25.51	−2.212	**0.029**
Psychological resilience score	67.58 ± 14.33	70.18 ± 14.39	60.69 ± 11.83	3.675	**<0.001**

The bold value represents statistically significant.

**Table 9D:** Analysis of influencing factors of GAD-7.

variable	Estimate	SE	*z*	Wald	*p*	OR (95% CI)
(Intercept)	2.272	0.962	2.361	5.572	**0.018**	
Psychological resilience score	−0.049	0.015	−3.358	11.276	**0.001**	0.952 (0.925, 0.980)

The bold value represents statistically significant.

**Table 9E:** Analysis of influencing factors of PHQ-9.

	Total (*n* = 142)	No depression (*n* = 78)	With depression (*n* = 64)	Statistical quantities	*p*
Psychological resilience score	67.58 ± 14.33	71.26 ± 12.92	63.09 ± 14.79	3.509	**0.001**

The bold value represents statistically significant.

**Table 9F:** Analysis of influencing factors of PHQ-9.

Variable	Estimate	SE	*z*	Wald	*p*	OR (95% CI)
(Intercept)	2.73	0.917	2.976	8.855	**0.003**	
Psychological resilience score	−0.044	0.013	−3.243	10.519	**0.001**	0.957 (0.933, 0.983)

The bold value represents statistically significant.

## Discussion

The global spread of the COVID-19 pandemic has raised concerns about the resulting mental health issues (Pfefferbaum and North, 2020). The pandemic is a major challenge for healthcare providers. Healthcare providers are facing significant challenges during the pandemic, especially in the particular working environment of the square cabin. Frontline healthcare providers are prone to various psychological and sleep-related problems due to the closed environment, increased risk of infection, high work intensity, and easy fatigue when working under protective equipment. Consistent with the results of related studies (Hou et al., 2020; Liang et al., 2020). However, the occurrence of anxiety and depression scores in this study was significantly lower than the characteristics of the control group, which indicates that although the incidence of anxiety and depression was higher among frontline healthcare workers in Makeshift hospitals, the symptoms of anxiety and depression were milder, which may be attributed to the long-term regular management of the pandemic and regular training organized by the hospital, where healthcare workers had some awareness and response to the pandemic and the effects of anxiety and depression were reduced to a lower level. The incidence and scores of insomnia were higher among frontline healthcare providers in the square cabin hospital than among other healthcare providers, which needed to be taken seriously. The analysis of influencing factors showed that age and psychological flexibility were the main factors of insomnia. Healthcare providers under 40 years old were more likely to have insomnia. These findings are consistent with previous studies. Indeed, the causes of psychological problems in frontline healthcare providers are complex. For example, during work in a square cabin hospital, there are long hours of intense work, tight staffing, a high risk of infection, isolation from family members in a challenging environment, etc. (Lai et al., 2020; Qiu et al., 2021). This leads to a series of discomforts for frontline medical staff. The high workload leads to the fact that when negative emotions are present and physical symptoms appear, the medical staff does not have time to fully deal with their own physical and mental discomforts to recover appropriately. The continued negative impact further aggravates their physical and psychological burden. With the continuing pandemic being managed regularly, practical and effective coping strategies must be implemented, such as continuing to strengthen the management of epidemic prevention and control, organizing regular training sessions, improving the coping ability and psychological tolerance of medical staff, and selecting team members with experience in epidemic prevention and control, to reduce the negative impact on frontline medical staff in their anti-epidemic work.

Psychological resilience helps people resist stressors and respond positively to adversity and is the dynamic process by which people successfully cope and adapt when experiencing hardship, trauma, or stress (Green et al., 2010; Poole et al., 2017). Controllability is essential in stressful situations and can help individuals cope with stressful situations (Vinkers et al., 2020). The less psychologically resilient an individual is, the higher the risk of developing anxiety and depression (Petzold et al., 2020). This study showed that the psychological resilience scores of frontline healthcare providers were not statistically different from those of the control medical staff, and anxiety and depression scores were low. Our findings were inconsistent with the results of some studies (Lai et al., 2020). In this study, we analyzed the reasons for this. First, healthcare providers working in the square cabin hospitals were well prepared behaviorally, psychologically, and spiritually under the pandemic normalization. Therefore, their psychological resilience is not much different from other healthcare providers. Most of the frontline healthcare providers in this survey who participated in Makeshift hospital were actively enrolled and voluntarily participated in the work of fighting the pandemic. Thus, those participants’ anxiety and depression scores were lower with the same psychological resilience score. In addition, we found that the healthcare providers who experienced insomnia, anxiety, and depression had significantly lower psychological resilience scores, suggesting that psychological resilience may help maintain mental health and help medical workers better recover from trauma to reduce the occurrence of insomnia, anxiety, and depression. These findings were consistent with Lin et al. (2020) findings. They explained that healthcare providers with higher psychological resilience scores might have a lower level of anxiety and depression in the face of public health emergencies. Psychological resilience training through medical practice can improve the ability of medical personnel to cope with an outbreak (Aiello et al., 2011). In addition, studies have shown that high levels of social support can prevent the adverse effects of low psychological resilience on mental health (Li et al., 2021), suggesting the importance of frontline medical staff in maintaining social connections and support. Research shows that supportive leadership can help healthcare professionals build psychological capital (Farid et al., 2021; Khattak et al., 2021). Positive mindfulness (Hossain and Clatty, 2021), cognitive behavioral (Zhao et al., 2020), narrative, and acceptance commitment therapies are effective methods to enhance psychological strength (Burton et al., 2010). Therefore, according to the psychological characteristics of frontline medical staff, it is of great practical significance to further explore psychological interventions from multiple aspects based on the results of previous relevant studies to improve the psychological resilience of medical staff and safeguard their mental health.

The healthcare providers in the square cabin hospital are anti-epidemic personnel from all over the country, and the frontline healthcare providers operate across the region, which will break the normal working and living conditions. The survey showed that frontline healthcare providers generally had multiple adverse somatic symptom reactions. The high incidence of dry eyes may be related to the prolonged wearing of goggles, which makes it difficult to adjust the eyes. The headache is related to the tightness of the goggle fixation belt in working conditions. The itchy skin may be related to dryness due to repeated skin decontamination for a long time and the decrease in immunity. The higher incidence rate of lumbar muscle strain may be related to the inability to put on and take off protective clothing and the obstruction of body movement in the protective clothing condition. Prolonged exposure to environments with high intensity, high risk of infection, high stress, disrupted work and rest, and various types of emergencies can easily cause problems in the sociopsychological protection system among healthcare providers. The issues in the sociopsychological protection system may finally cause stress injuries. Our study suggests that healthcare providers should exclude those suffering from abnormalities in ventilation, diffusion function, or other related underlying diseases because the workload in the square cabin is high. After entering the frontline work, understanding the specific situation of frontline healthcare providers and taking targeted measures may reduce the adverse reactions among healthcare providers. For example, When forming the anti-epidemic team, pay attention to the matching of age levels, give priority to medical personnel with anti-epidemic experience, reduce the workload in policy, enhance immunity, give appropriate intramuscular injections of thymidine, maintain the self-health maintenance of medical personnel, pay attention to the adjustment of protective equipment such as masks, goggles, and protective clothing so that the body is at a relatively comfortable and acceptable level before entering the red zone and after leaving it at the end of work. They must drink an appropriate amount of water, pay attention to the intensity of work, reduce the intensity of work activities, and meet the protective requirements to put on and take off protective clothing. Meanwhile, medical personnel should promptly assess and deal with physical and mental symptoms, ensuring adequate rest and recovery and minimizing the impact of various symptoms on medical personnel.

## Conclusion

In summary, a survey of the mental and sleep status of frontline medical staff and medical personnel in the cabin in the pandemic environment revealed that frontline medical staff’s physical and mental health deserved attention. Somatic symptoms were more prevalent among frontline medical staff during the fight against the pandemic. The prevalence of anxiety, depression, and insomnia was higher than in others. However, the level of anxiety and depression has decreased, and the adverse effects remain within a reasonable range. The standing pandemic management policy has provided frontline medical staff with good psychological resilience and coping measures to effectively handle the psychological challenges and impacts of frontline work. However, somatization and insomnia symptoms are more severe and may negatively impact physical health. Necessary interventions and pharmacological strategies can be considered, which will positively impact the treatment and prognosis of medical staff during a pandemic.

Our study has limitations in using a homemade symptom observation scale. Cross-sectional studies cannot respond to changes over time reflecting the long-term effects and characteristics of pandemic work on frontline medical personnel’s physical and mental health. In contrast, the characteristics of body shape changes may vary among different populations in different geographic regions. It is necessary to expand the sample size for a multi-stage survey, a part of this study that could not be conducted. In addition, the relevant interventions are yet to be tested for their practical effects in further studies.

## Data availability statement

The raw data supporting the conclusions of this article will be made available by the authors, without undue reservation.

## Ethics statement

The studies involving human participants were reviewed and approved by the Ethics Committee of the Army Specialty Medical Center. The patients/participants provided their written informed consent to participate in this study.

## Author contributions

HY and LZ contributed to the conception and design of the study. FG, QW, ZL, and PL conducted the questionnaire administration and data collection. HP and QW conducted the statistical analysis. HP wrote the first draft of the manuscript. XG, FG, QW, and DL wrote parts of the manuscript. All authors participated in the revision of the manuscript and read and approved the submitted version.

## Funding

This research was supported by the Special Project of Technological Innovation and Application Development of Chongqing, China (Grant NO. cstc2019jscx-msxmX0248).

## Conflict of interest

The authors declare that the research was conducted in the absence of any commercial or financial relationships that could be construed as a potential conflict of interest.

## Publisher’s note

All claims expressed in this article are solely those of the authors and do not necessarily represent those of their affiliated organizations, or those of the publisher, the editors and the reviewers. Any product that may be evaluated in this article, or claim that may be made by its manufacturer, is not guaranteed or endorsed by the publisher.

## References

[ref1] Aiello A. Khayeri M. Y. Raja S. Peladeau N. Romano D. Leszcz M.. (2011). Resilience training for hospital workers in anticipation of an influenza pandemic. J. Contin. Educ. Heal. Prof. 31, 15–20. doi: 10.1002/chp.20096, PMID: 21425355

[ref2] Brainin P. Biering-Sorensen S. R. Mogelvang R. Sogaard P. Jensen J. S. Biering-Sorensen T. (2018). Postsystolic shortening by speckle tracking echocardiography is an independent predictor of cardiovascular events and mortality in the general population. J. Am. Heart Assoc. 7. doi: 10.1161/JAHA.117.008367, PMID: 29519813 PMC5907576

[ref3] Burton N. W. Pakenham K. I. Brown W. J. (2010). Feasibility and effectiveness of psychosocial resilience training: a pilot study of the READY program. Psychol. Health Med. 15, 266–277. doi: 10.1080/13548501003758710, PMID: 20480432

[ref4] Cheng C. Dong D. He J. Zhong X. Yao S. (2020). Psychometric properties of the 10-item Connor-Davidson resilience scale (CD-RISC-10) in Chinese undergraduates and depressive patients. J. Affect. Disord. 261, 211–220. doi: 10.1016/j.jad.2019.10.018, PMID: 31654919

[ref5] Farid T. Iqbal S. Saeed I. Irfan S. Akhtar T. (2021). Impact of supportive leadership during Covid-19 on nurses' well-being: the mediating role of psychological capital. Front. Psychol. 12:695091. doi: 10.3389/fpsyg.2021.695091 34659011 PMC8515951

[ref6] Green K. T. Calhoun P. S. Dennis M. F. Beckham J. C. (2010). Exploration of the resilience construct in posttraumatic stress disorder severity and functional correlates in military combat veterans who have served since September 11th, 2001. J. Clin. Psychiatry 71, 823–830. doi: 10.4088/JCP.09m05780blu, PMID: 20584523

[ref7] Hossain F. Clatty A. (2021). Self-care strategies in response to nurses moral injury during COVID-19 pandemic. Nurs. Ethics 28, 23–32. doi: 10.1177/0969733020961825, PMID: 33124492 PMC7604672

[ref8] Hou T. Zhang T. Cai W. Song X. Chen A. Deng G.. (2020). Social support and mental health among health care workers during coronavirus disease 2019 outbreak: a moderated mediation model. PLoS One 15:e0233831. doi: 10.1371/journal.pone.0233831, PMID: 32470007 PMC7259684

[ref9] Khattak S. R. Saeed I. Rehman S. U. Fayaz M. (2021). Impact of fear of COVID-19 pandemic on the mental health of nurses in Pakistan. J. Loss Trauma 26, 421–435. doi: 10.1080/15325024.2020.1814580, PMID: 36160588

[ref10] Kroenke K. Spitzer R. L. Williams J. B. (2001). The PHQ-9: validity of a brief depression severity measure. J. Gen. Intern. Med. 16, 606–613. doi: 10.1046/j.1525-1497.2001.016009606.x, PMID: 11556941 PMC1495268

[ref11] Lai J. Ma S. Wang Y. Cai Z. Hu J. Wei N.. (2020). Factors associated with mental health outcomes among health care workers exposed to coronavirus disease 2019. JAMA Netw. Open 3:e203976. doi: 10.1001/jamanetworkopen.2020.3976, PMID: 32202646 PMC7090843

[ref12] Li F. Luo S. Mu W. Li Y. Ye L. Zheng X.. (2021). Effects of sources of social support and resilience on the mental health of different age groups during the COVID-19 pandemic. BMC Psychiatry 21, 1–14. doi: 10.1186/s12888-020-03012-1 33413238 PMC7789076

[ref13] Li H. Zhang Y. Li Y. Wang Z. (2019). Application and practice of “questionnaire star” in C language flipped classroom teaching. Heilongjiang. Sci. 10, 22–23.

[ref14] Liang Y. Wu K. Zhou Y. Huang X. Zhou Y. Liu Z. (2020). Mental health in frontline medical workers during the 2019 novel coronavirus disease epidemic in China: a comparison with the general population. Int. J. Environ. Res. Public Health 17:6550. doi: 10.3390/ijerph17186550, PMID: 32916836 PMC7558595

[ref15] Lin J. Ren Y. H. Gan H. J. Chen Y. Huang Y. F. You X. M. (2020). Factors associated with resilience among non-local medical workers sent to Wuhan, China during the COVID-19 outbreak. BMC Psychiatry 20:417. doi: 10.1186/s12888-020-02821-8, PMID: 32831045 PMC7443813

[ref16] Luo M. Guo L. Yu M. Jiang W. Wang H. (2020). The psychological and mental impact of coronavirus disease 2019 (COVID-19) on medical staff and general public–a systematic review and metaanalysis. Psychiatry Res. 291:113190. doi: 10.1016/j.psychres.2020.113190, PMID: 32563745 PMC7276119

[ref17] Morin C. M. Belleville G. Bélanger L. Ivers H. (2011). The insomnia severity index: psychometric indicators to detect insomnia cases and evaluate treatment response. Sleep 34, 601–608. doi: 10.1093/sleep/34.5.601, PMID: 21532953 PMC3079939

[ref18] Petzold M. B. Bendau A. Plag J. Pyrkosch L. Mascarell Maricic L. Betzler F.. (2020). Risk, resilience, psychological distress, and anxiety at the beginning of the COVID-19 pandemic in Germany. Brain Behav. 10:e01745. doi: 10.1002/brb3.1745, PMID: 32633464 PMC7361063

[ref19] Pfefferbaum B. North C. S. (2020). Mental health and the Covid-19 pandemic. N. Engl. J. Med. 383, 510–512. doi: 10.1056/NEJMp2008017, PMID: 32283003

[ref20] Plummer F. Manea L. Trepel D. McMillan D. (2016). Screening for anxiety disorders with the GAD-7 and GAD-2: a systematic review and diagnostic metaanalysis. Gen. Hosp. Psychiatry 39, 24–31. doi: 10.1016/j.genhosppsych.2015.11.005, PMID: 26719105

[ref21] Poole J. C. Dobson K. S. Pusch D. (2017). Childhood adversity and adult depression: the protective role of psychological resilience. Child Abuse Negl. 64, 89–100. doi: 10.1016/j.chiabu.2016.12.012, PMID: 28056359

[ref22] Qiu Y. Wu Q. Chen R. Guan C. (2021). Research on psychological stress and mental health of medical staff in COVID-19 prevention and control. Int. J. Disaster Risk Reduct. 65:102524. doi: 10.1016/j.ijdrr.2021.102524, PMID: 34458085 PMC8379899

[ref23] Rahman M. A. Islam S. M. S. Tungpunkom P. Sultana F. Alif S. M. Banik B.. (2021). COVID-19: factors associated with psychological distress, fear, and coping strategies among community members across 17 countries. Glob. Health 17, 1–19. doi: 10.1186/s12992-021-00768-3 PMC8485312 34598720

[ref24] Spitzer R. L. Kroenke K. Williams J. B. Löwe B. (2006). A brief measure for assessing generalized anxiety disorder: the GAD-7. Arch. Intern. Med. 166, 1092–1097. doi: 10.1001/archinte.166.10.1092, PMID: 16717171

[ref25] Tawfik D. S. Scheid A. Profit J. Shanafelt T. Trockel M. Adair K. C.. (2019). Evidence relating health care provider burnout and quality of care: a systematic review and metaanalysis. Ann. Intern. Med. 171, 555–567. doi: 10.7326/M19-1152, PMID: 31590181 PMC7138707

[ref26] Vinkers C. H. van Amelsvoort T. Bisson J. I. Branchi I. Cryan J. F. Domschke K.. (2020). Stress resilience during the coronavirus pandemic. Eur. Neuropsychopharmacol. 35, 12–16. doi: 10.1016/j.euroneuro.2020.05.003, PMID: 32446705 PMC7211573

[ref27] Wang W. Bian Q. Zhao Y. Li X. Wang W. Du J.. (2014). Reliability and validity of the Chinese version of the patient health questionnaire (PHQ-9) in the general population. Gen. Hosp. Psychiatry 36, 539–544. doi: 10.1016/j.genhosppsych.2014.05.021, PMID: 25023953

[ref28] Wu Q. Li D. Yan M. Li Y. (2022). Mental health status of medical staff in Xinjiang Province of China based on the normalization of COVID-19 epidemic prevention and control. Int. J. Disaster Risk Reduct. 74:102928. doi: 10.1016/j.ijdrr.2022.102928, PMID: 35368428 PMC8958729

[ref29] Zhang D. Luo H. Xiao L. Zhang Z. Huang J. Li X.. (2022). Depression and insomnia of front-line medical staff during the COVID-19 outbreak in China: an online Cross-sectional study. Front. Psychol. 13:897896. doi: 10.3389/fpsyg.2022.897896, PMID: 35846703 PMC9277440

[ref30] Zhang W. R. Wang K. Yin L. Zhao W. F. Xue Q. Peng M.. (2020). Mental health and psychosocial problems of medical health workers during the COVID-19 epidemic in China. Psychother. Psychosom. 89, 242–250. doi: 10.1159/000507639, PMID: 32272480 PMC7206349

[ref31] Zhao X. Zhang T. Li B. Yu X. Ma Z. Cao L.. (2020). Job-related factors associated with changes in sleep quality among healthcare workers screening for 2019 novel coronavirus infection: a longitudinal study. Sleep Med. 75, 21–26. doi: 10.1016/j.sleep.2020.07.027, PMID: 32853914 PMC7403128

